# Assessing the risk factors of cholera epidemic in the Buea Health District of Cameroon

**DOI:** 10.1186/s12889-015-2485-8

**Published:** 2015-11-14

**Authors:** Dickson Shey Nsagha, Julius Atashili, Peter Nde Fon, Elvis Asangbeng Tanue, Charlotte Wenze Ayima, Odette Dzemo Kibu

**Affiliations:** Department of Public Health and Hygiene, Faculty of Health Sciences, University of Buea, P.O Box 12, Buea, Cameroon; Department of Medical Laboratory Sciences, Faculty of Health Sciences, University of Buea, P.O Box 12, Buea, Cameroon

**Keywords:** Cholera, Risk factors, Epidemic, Attack rate, Cameroon

## Abstract

**Background:**

Cholera is an acute diarrheal disease caused by the bacterium, *Vibrio cholerae*. A cholera epidemic occurred in Cameroon in 2010. After a cholera-free period at the end of 2010, new cases started appearing in early 2011. The disease affected 23,152 people and killed 843, with the South West Region registering 336 cases and 13 deaths. Hence, we assessed the risk factors of cholera epidemic in the Buea Health District to provide evidence-based cholera guidelines.

**Methods:**

We conducted an unmatched case–control study. Cases were identified from health facility records and controls were neighbours of the cases in the same community. We interviewed 135 participants on socio-economic, household hygiene, food and water exposures practices using a semi-structured questionnaire. Data was analyzed using STATA. Fisher exact test and logistic regression were computed. *P* < 0.05 was considered to be statistically significant.

**Results:**

The 135 participants included 34 (25.2 %) cholera cases and 101 (74.8 %) controls. More females [78 (57.8 %)] participated in the study. Ages ranged from 1 year 3 months to 72 years; with a mean of 29.86 (±14.51) years. The cholera attack rate was 0.03 % with no fatality. Most participants [129 (99.2 %)] had heard of cholera. Poor hygienic practices [77 (59.2 %)] and contaminated water sources [54 (41.5 %)] were the main reported transmission routes of cholera. Good hygienic practices [108 (83.1 %)] were the main preventive methods of cholera in both cases [23 (76.6 %)] and controls [85 (85.0 %)]. Logistic regression analysis showed age below 21 years (OR = 1.72, 95 % CI: 0.73–4.06, *p* = 0.251), eating outside the home (OR = 1.06, CI: 0.46–2.43, *p* = 1.00) and poor food preservation method (OR = 9.20, CI: 3.67–23.08, *p* < 0.0001) were independent risk factors of cholera. Also, irregular water supply (OR = 0.66, 95 % CI: 0.30–1.43, *p* = 0.320), poor kitchen facility (OR = 0.60, CI: 0.16–2.23, *p* = 0.560), lack of home toilet (OR = 0.69, CI: 0.25–1.86, *p* = 0.490), and education below tertiary (OR = 0.87, 95 % CI: 0.36–2.11, *p* = 0.818) were independent protective factors for the occurrence of cholera.

**Conclusion:**

There was a good knowledge of cholera among participants. Poor food preservation method was a significant independent risk factor of cholera. Improvement in hygiene and sanitation conditions and water infrastructural development is crucial to combating the epidemic.

## Background

Cholera is an acute diarrheal disease caused by the bacterium, *Vibrio cholerae*; an infection in the intestines that can kill even a healthy adult in a matter of hours [1]. As from 2000, the incidence of cholera has increased steadily, culminating in 317,534 reported cases worldwide, including 7543 deaths with a case-fatality rate of 2.38 % in 2010 [2]. The disease is now considered to be endemic in many countries and the pathogen causing cholera cannot currently be eliminated from the environment [3]. Regions of the world where Cholera is currently prevalent are Africa, Asia and parts of the Middle East. Sub-Saharan Africa is broadly affected by many cholera epidemics [4]. In Cameroon, the burden of cholera has increased during the past two decades. The annual number of reported cases had increased over the years [2] with 4026 cases in 1991, 5796 in 1996, 8005 in 2004 [5] and 10,759 in 2010 [6]. Cholera can spread rapidly through a population resulting in individuals with dehydration and causing severe morbidity and mortality [7]. The infection is transmitted through contaminated fecal matter, which can be consumed through tainted food and water sources or because of poor hygiene and sanitation, like unwashed hands [8]. Cholera is most common in areas that lack clean water sources and sanitation services. Areas like refugee camps and urban slums, where people live in close proximity with little to no access to clean water and sanitation facilities are at a very high risk of experiencing a cholera epidemic [8].

Access to potable water in both rural and urban centers of Cameroon is a great concern [9, 10]. A study carried out by Jane-Francis and colleagues [11] in Douala, Cameron reported wells as reservoirs of *V. cholerae.* However, risk factors for cholera in Cameroon have not been evaluated systematically. Numerous possible explanations for the current outbreak exist, including poor hygiene and sanitation and environmental factors [12]. A majority of cholera epidemics and deaths have been reported in sub-Saharan Africa [6] where the risk of cholera infection is high. Typical at-risk areas include peri-urban slums where basic infrastructure is not available and camps for internally displaced people where the minimum requirements of clean water and sanitation are not met [13]. The greatest risk occurs in over-populated communities and refuge settings characterized by poor sanitation, unsafe drinking water and increased person to person transmission [14]. The 2010 cholera epidemic in Cameroon affected 10,741 people and killed 650. An ever increasing number of cholera cases were registered almost everywhere in the first week of 2011 in Cameroon. The South West Region of Cameroon was affected by this epidemic. Hence, it was necessary to assess the risk of cholera in the Buea Health District and to provide evidence-based cholera epidemic guidelines.

## Methods

### Study area

This study was conducted in the Buea Health District in the South West Region of Cameroon in 2011. Buea is situated at 4.15° North latitude, 9.24° East longitude in the Fako-Division. It has a population of about 200,000 inhabitants and is located 15 km from the Atlantic Ocean and 60 km from Douala, the economic capital of Cameroon [15]. There are many ethnic groups in Buea including the Bakweri (indigenes), Bamileke, Bafou, Balondu, Metta and Bayangi among others. Most inhabitants practice agriculture as the main economic activity [15]. Almost all ethnic groups in Cameroon are represented in Buea, attracted by the fertile volcanic soil and the Cameroon Development Corporation, a giant agricultural Corporation that seconds the state of Cameroon in employment [15]. Access to portable water is a major problem in Buea where power supply is erratic.

### Study design

This was an unmatched case–control study in the Buea Health District. Cases were identified in hospitals and health facilities and a follow up of their neighborhood contacts was conducted and recruited as controls in their communities. Cases and controls exposure histories were compared for evaluation of risk factors during the outbreak using a semi-structured questionnaire. The study was conducted based on a request from the Cameroon Ministry of Public Health to the Department of Public Health and Hygiene of the University of Buea because of their epidemic.

### Participants

#### Selection of cases

Patient admission logbooks at the hospital and health facilities were reviewed to identify persons admitted for diarrhea. Patients who met the clinical case definition for cholera as defined by the Ministry of Public Health were enrolled for the study. A cholera case was any patient without discrimination of age hospitalized for acute diarrhea with/without vomiting during the cholera epidemic. Cases were identified without knowledge of the vaccination status.

#### Selection of controls

Two unmatched controls per case were recruited from the residential area of the cases. Persons living in the same neighborhood with the cases and had non-diarrheal conditions were recruited as controls in their communities. Controls were chosen without being unaware of their vaccination status.

### Administrative clearance

Administrative authorization was obtained from the Regional Delegation of the Ministry of Public Health for the South West Region. District Health Services were contacted for approval.

### Ethical considerations

The study qualified for exemption from ethical review as it was conducted as part of an outbreak investigation to identify the risk factors of cholera. Verbal consent was obtained in place of written consent from both cases and controls.

### Study procedures

#### Data collection

The study was conducted from March to August 2011. Data were obtained through review of clinical records and participant interviews using a semi-structured questionnaire. Demographic characteristics such as age, gender, level of education, occupation and behavioral characteristic such as hand washing, hygiene and sanitation (toilet with flush, latrine, none) and exposure factors were collected from participants.

#### Data management and analysis

Data were entered into Microsoft Excel for cleaning. At the initial step of the data analysis, frequency distributions of each variable were produced. Associations were established between variables of different measures through cross-tabulations. Logistic regression and Fisher exact test were used to test the significance of associations between categorical variables using STATA version 10 (Stata ORp., College Station, TX, USA). Statistical significance was set at *p* < 0.05 at 95 % confidence level.

#### Limitations of the study

Recall bias was not a problem because the study took place immediately after the epidemic. The selection of unmatched controls from the same neighborhood as the cases was a major limitation of the study. However, choosing controls from the same neighborhood with cases controlled for socio-demographic factors. The reliability of participant’s response was not assessed. Also, the cholera prevention messages disseminated by the Ministry of Public Health during the cholera outbreak might have affected the responses of participants.

## Results

### General demographic characteristics of study participants

**Fig. 1:**
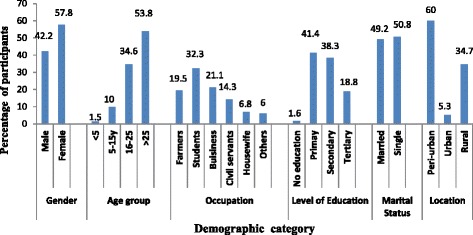
General demographic characteristics of Study Participants

Among the 135 participants, 57 (42.2 %) were males and 78 (57.8 %) were females. The ages ranged from 1 year 3 months to 72 years with the mean age being 29.86 ± 14.51 years. Most participants were above 25 years of age (Fig. 1).

### Demographic characteristics among study participants by cholera cases and non- cases

The 135 participants included 34 (25.2 %) cholera cases and 101 (74.8 %) controls. The females participated in the study included [20 (58.2 %)] cholera cases and [58 (57.4 %)] controls. No fatality was recorded from the data collected during this outbreak investigation with an attack rate of 0.03 %. Most of the participants [75 (60.0 %)] were from the peri-urban areas of the health district with Bolifamba village having the highest number of cholera cases [9 (33.3 %)] (Table 1).

**Table 1:** Demographic characteristics of study participants based on cholera cases and controls

Characteristics	Cases (*n* = 34)	Controls (*n* = 101)	Total (*n* = 135)	*χ* ^2^	*p*-value
	No (%)	No (%)	No (%)		
Gender					
Male	14 (41.2)	43 (42.6)	57 (42.2)	0.020	0.886
Female	20 (58.2)	58 (57.4)	78 (57.8)		
Age group					
< 5	0.2 (6.3)	0 (0)	02 (1.5)	6.645	0.084
5–15	12.5 (12.5)	9 (9.2)	13 (10.0)		
16–25	10 (31.3)	35 (35.7)	45 (34.6)		
> 25	16 (50.0)	54 (55.1)	70 (53.8)		
Occupation					
Farmers	5 (15.5)	21 (21.0)	26 (19.5)	5.849	0.321
Students	14 (42.5)	29 (29.0)	43 (32.3)		
Business	8 (24.2)	20 (20.0)	28 (21.1)		
Civil servant	3 (9.1)	16 (16.0)	19 (14.3)		
Housewife	3 (9.1)	6 (6.0)	9 (6.8)		
Others	0 (0.0)	8 (8.0)	8 (6.0)		
Level of Education					
No formal education	0 (0.0)	2 (2.1)	2 (1.6)	1.516	0.678
Primary	15 (48.4)	38 (39.2)	53 (41.4)		
Secondary	10 (32.3)	39 (40.2)	49 (38.3)		
Tertiary	6 (19.4)	18 (18.6)	24 (18.8)		
Marital Status					
Married	14 (45.2)	49 (50.5)	63 (49.2)	0.269	0.603
Single	17 (54.8)	48 (49.5)	65 (50.8)		
Location					
Peri-urban	16 (57.1)	41 (61.2)	57 (60)	2.372	0.305
Urban	03 (10.7)	02 (3.0)	05 (5.3)		
Rural	09 (32.1)	24 (35.8)	33 (34.7)		

### Assessment on the knowledge of cholera among study participants

Most participants [129 (95.6 %)] reported to have heard of the cholera epidemic. The media [85 (62.9 %)] and local population [61 (45.2 %)] were the main reported sources of cholera information. Thirty (88.2 %) of the cases had heard of cholera before. Poor hygienic practices [77 (57.0 %)] and lack of safe drinking water [49 (36.3 %)] were the main conveyed risk factors of cholera with 17 (50.0 %) and 11 (32.4 %) of these infected with the disease. Many respondents mentioned that cholera can be spread through contaminated water [54 (40.0 %)]. However, respondents who indicated contaminated food [13 (38.2 %)] as the source of infection were equally infected with cholera. Most respondents [108 (80.0 %] indicated good hygienic practices was the main cholera preventive method (Table 2). No participant specifically mentioned *Vibrio cholerae* as the causative agent of cholera.

**Table 2:** Knowledge of cholera among study participants

Characteristics	Cases (*n* = 34)	Controls (*n* = 101)	Total (*n* = 135)	*χ* ^2^	*p*-value
	No (%)	No (%)	No (%)		
Heard of Cholera					
Yes	30 (88.2)	99 (98.0)	129 (95.6)	3.662	0.056
No	04 (11.8)	02 (2.0)	06 (4.4)		
Source of information					
Media	16 (47.1)	69 (68.3)	85 (62.9)	2.161	0.339
Local population	17 (50)	44 (43.5)	61 (45.2)		
Hospital	03 (8.8)	16 (15.8)	19 (14.1)		
Risk factors of Cholera					
Lack of safe drinking-water	11(32.4)	38 (37.6)	49 (36.3)	4.995	0.288
Eating rotten food/fruits	03 (8.8)	29 (28.7)	32 (25.7)		
Poor hygiene practices	17 (50)	60 (59.4)	77 (57.0)		
Infected by cholera germ	03 (8.8)	08 (8.0)	12 (8.9)		
No idea	03 (2.8)	04 (3.9)	07 (5.2)		
Transmission of Cholera					
Contaminated food	13 (38.2)	27 (26.7)	40 (29.6)	3.142	0.534
Contaminated water	10 (29.4)	44 (43.6)	54 (40.0)		
Poor hygiene and sanitation	07 (20.6)	29 (28.7)	36 (26.7)		
Contact with infected persons	06 (17.6)	22 (21.8)	28 (20.7)		
No idea	05 (14.7)	13 (12.9)	18 (13.3)		
Prevention of Cholera					
Good hygienic practices	23 (67.6)	85 (84.2)	108 (80.0)	7.887	0.096
Avoiding infected persons	02 (5.9)	02 (2.0)	04 (3.0)		
Water treatment	05 (14.7)	09 (8.9)	14 (10.3)		
Drink potable water	07 (20.6)	35 (34.7)	42 (31.1)		
No idea	03 (8.8)	02 (2.0)	05 (3.7)		

### Risk factors of cholera

Majority of the participants [126 (95.5 %)] used tap water as the main source of drinking water. The use of springs as the main source of drinking water was an independent protective factor of cholera infection (OR: 0.58, 95 % CI: 0.07–5.16, *p* = 1.0000). The problem of irregular water supply was most frequent [78 (58.6 %)] among tap water users with the odds of exposure for cholera cases being 0.66 times the odds of exposure in those without the disease. Most participants [97 (74.0 %)] had home toilet facilities with cholera cases 0.69 times more likely to be without home toilet facilities than those without the disease (OR: 0.69, 95 % CI: 0.25–1.86, *p* = 0.490). Poor kitchen facility was an independent protective factor of cholera infection (OR: 0.60, 95 % CI: 0.16–2.23, *p* = 0.560) (Table 3).

**Table 3:** Risk factors of cholera in the study population

Risk Factors	Total	Cases (*n* = 34)		Controls (*n* = 101)		Odds Ratio	Confidence Interval	Fisher exact *p*-value
	No (%)	No (%)	Odds of exposure	No (%)	Odds of exposure		95 % CI	
Spring as drinking water source	6 (4.5)	01 (3.0)	0.03	05 (5.0)	0.05	0.58	0.07–5.16	1.00
Irregular water supply	78 (58.6)	17 (51.5)	1.00	61 (61.0)	1.53	0.66	0.30–1.43	0.32
Lack of home toilet facility	30 (22.9)	6 (19.4)	0.21	24 (24.0)	0.31	0.69	0.25–1.86	0.49
Poor kitchen facility	17 (31.2)	3 (10.0)	0.10	14 (14.1)	0.16	0.60	0.16–2.23	0.56
Poor food preservation method	29 (25.2)	18 (62.1)	1.13	11 (66.3)	0.12	9.20	3.67–23.08	<0.0001
Eating outside the home	90 (66.7)	23 (67.7)	2.09	67 (66.3)	1.97	1.06	0.46–2.43	1.00
Below 21 years of age	33 (25.4)	11 (34.4)	0.48	22 (22.5)	0.28	1.72	0.73–4.06	0.25
Education below tertiary level	102 (80.3)	25 (80.7)	2.78	77 (81.1)	3.21	0.87	0.36–2.11	0.82

Most participants [86 (74.78 %)] practiced good food preservation methods. Moreover, many [90 (66.67 %)] ate outside their homes such as in restaurants and from road site food vendors (Table 2). Those with cholera were 9.2 times more likely to practice poor food preservation methods than those without the disease (OR: 9.20, 95 % CI: 3.67–23.03, *p* < 0.0001). An equal odds of cholera infection was observed between cases and controls who ate outside the home (OR: 1.06, CI: 0.46–2.43, *p* = 1.00).

More of the participants below 21 years of age [11 (34.3 %)] were infected with cholera compared to those 21 years and above. Those with cholera were 72 % more likely to be below the age of 21 years than those without the disease (OR: 1.72, 95 % CI: 0.73–4.06, *p* = 0.251). Participants with cholera were 0.87 times likely to have a below tertiary level of education than controls (OR: 0.87, 95 % CI: 0.36–2.11, *p* = 0.818).

## Discussion

Cholera continues to be a global threat to public health and a key indicator of lack of social development. Once common throughout the world, the infection is now largely confined to developing countries in the tropics and subtropics [16]. For a cholera outbreak to occur, two conditions have to be met: there must be significant breaches in the water, sanitation, and hygiene infrastructure used by groups of people, permitting large-scale exposure to food or water contaminated with *Vibrio cholera*e organisms; and cholera must be present in the population [17]. Improving water, sanitation and other infrastructure has been associated with a 39 % decline in waterborne disease in informal urban settlements in Africa [18]. It was necessary to determine a population specific level of awareness and risk factors of cholera infection in the country.

A number of demographic and socioeconomic factors including age, gender and social status are also known to play a crucial role to cholera infection. There were more females [20 (58.2 %)] cholera cases than males [14 (41.2 %)]. Most women are engaged in domestic activities which expose them to this infection. Cholera was mostly reported from students [14 (42.5 %)] than from other occupations. The Buea Health District is a cosmopolitan locality with people from various parts of the nation for academic purposes because of the presence of the University of Buea. The proportion of respondents with good knowledge of cholera was very high probably because of the cholera prevention programme from the Ministry of Public Health. This finding is consistent with published data from Peru [16] which showed a high knowledge of cholera and that cholera prevention campaign successfully educated respondents. However, our findings were contrary to a Tanzanian cross-sectional survey conducted to assess knowledge, attitudes and practices [19] in which the level of knowledge concerning cholera was very low. Analysis of knowledge levels compared to social, hygienic and personal practices showed that respondents obtained information about cholera mostly from the media [85 (65.4 %)] compared to health facilities. This may be because in most cholera-endemic communities, the governments use health education through mass media as the major preventive method against cholera. The strategy is to create awareness of the existence of the disease and also provide the population with the basic knowledge in first aid to handle cases. However, in our study area, a lot of sensitization is conducted to improve sanitary conditions in homes. Poor hygienic practices and contaminated water sources were the main transmission routes of cholera. Improving infrastructural, social, behavioral and personal hygiene and sanitation are the cornerstone of cholera prevention and have been shown to dramatically lessen the impact of epidemics [20, 21]. From our study, poor food preservation methods, eating outside the home and below 21 years of age were independent risk factors of the cholera epidemic. High cholera mortality has been reported in both adults and children [2]. Another study by Siddique and colleagues [22] revealed that the proportion of severe dehydration among *V. cholerae*-infected children was significantly higher compared to the proportion of rotavirus-infected children. However, our study reported that most of the participants below 21 years old were infected with cholera compared to those 21 years and above.

From our study, those with cholera were about 9 times more likely to practice poor food preservation methods than those without the disease. This is in line with an American study which showed that the cause of cholera from the U.S coastal waters was due to the consumption of raw, uncooked or contaminated shell fish [23].

Lack of potable water and irregular water supply were independent protective factors of cholera. There is ample evidence of the importance of water quality from the Mexican 1991 epidemic [24]. Contaminated water sources and the resultant water quality were found to be the most common causes for cholera in separate studies in Peru, Mexico and Ecuador [25, 26]. However, the government of Cameroon has been building more potable water points to salvage this situation.

The lack of access to latrines has also been identified as a risk factor for cholera in informal settlement areas [27]. In a study carried out by Alexander and colleagues [28], cases of cholera were more likely to defecate in the open air or river than controls. Contrary to our results, cases that defecated in bushes and rivers (lack of home toilets) had equal odds of being infected with cholera compared to participants without the disease. Our finding is in line with those reported by Ali and colleagues [29], Colobara and colleagues [30], and Stacie and colleagues [31] where increasing educational levels and decreasing cholera hospitalization risk have been reported to be associated. Also, Alexander and colleagues [28] reported that, higher levels of education were correlated with reduced risk for cholera hospitalization in both rural and urban Bangladesh. Significantly, most participants who were infected with cholera practiced poor food preservation methods. This is similar to an American study on cholera epidemic [31]. However, our study showed that participants with a tertiary level of education were equally likely to be infected with cholera compared to those who were less educated.

## Conclusion

There was a good knowledge of cholera among participants which is very important for prevention and control. Cholera risk factors were higher among the cases. Poor food preservation methods, eating from road side food vendors and below 21 years of age were independent risk factors for the occurrence of cholera. Improvement in hygiene and sanitation conditions coupled with water infrastructural development is crucial to combating the cholera epidemic.

## References

[1] World Health Organization. Cholera (2014). Available at: http://www.who.int/entity/wer/2015/wer9040.pdf?ua=1. Accessed October 31, 2014.

[2] Mohammad A, Anna LL, Young AY, Young EK, Binod S, Brian M (2012). The global burden of cholera. Bull World Health Organ.

[3] World Health Organization (2008). Outbreak news. Severe acute watery diarrhoea with cases positive for Vibrio cholerae, Viet Nam. Wkly Epidemiol Rec.

[4] Gaffga NH, Tauxe RV, Mintz ED (2007). Cholera: a new homeland in Africa?. Am J Trop Med Hyg.

[5] World Health Organization. Cholera in Cameroon. Available at: http://www.who.int/csr/don/2004_06_15/en. Accessed on 16^th^ June 2015.

[6] World Health Organization. Cholera fact sheet. Available at: http://www.who.int/mediacentre/factsheets/fs107/en/. Accessed 8^th^ January 2012.

[7] Griffith DC, Kelly-Hope LA, Miller MA (2006). Review of reported cholera outbreaks worldwide, 1995–2005. Am J Trop Med Hyg.

[8] International Medical Corps: Basic Facts on Cholera. Available at: https://internationalmedicalcorps.org/sslpage.aspx?pid=475. Accessed 19^th^ May 2015.

[9] Kuitcha D, Ndjama J, Tita AM, Lienou G, Kamgang KBV, Ateba BH (2010). Bacterial contamination of water points of the upper Mfoundi watershed, Yaounde, Cameroon. Afr J Microbiol Res.

[10] Ndjama J, Kamgang KBV, Sigha NL, Ekodeck GE, Awah TM (2008). Water supply, sanitation and health risks in Douala, Cameroon. Afr J Environ Sci Technol.

[11] Jane-Francis TKA, Christelle KP (2014). Water sources as reservoirs of Vibrio cholerae O1 and non-O1 strains in Bepanda, Douala (Cameroon): relationship between isolation and physico-chemical factors. BMC Infect Dis.

[12] Dempouo LD, Bradford DG, Ondobo GA, Etoundi GAM (2013). National surveillance data on the epidemiology of cholera in Cameroon. J Infect Dis.

[13] Swerdlow DL, Malenga G, Begkoyian G, Nyangulu D, Toole M, Waldman RJ (1997). Epidemic cholera among refugees in Malawi, Africa: treatment and transmission. Epidemiol Infect.

[14] Khonje A, Metcalf CA, Diggle E, Mlozowa D, Jere C, Akesson A (2012). Cholera outbreak in districts around Lake Chilwa, Malawi: Lessons learned. Malawi Med J.

[15] Nsagha D, Njunda A, Kamga H, Assob J, Bongkem E (2012). HIV-1/HIV-2 Co-infection among voluntary counseling testing subjects at a regional hospital in Cameroon. Afr J Health Sci.

[16] Ajoke OA, Solayide AA, Francisca ON, Mary-Theresa N, Akitoye OC (2012). Cholera epidemiology in Nigeria: an overview. Pan Afr Med J.

[17] Ecology Today. Health Organizations Attempt to Contain Cholera Outbreak in Haiti. 2015. Available at: http://www.ecology.com/2010/10/30/health-organizations-attempt-to-contain-cholera-outbreak-in-haiti. Accessed on 17^th^ July 2015.

[18] Sheuya SA (2008). Improving the health and lives of people living in slums. Ann N Y Acad Sci.

[19] Veronicaa MM, Kagoma SM (2005). Knowledge, attitudes and practices regrading cholera outbreaks in Ilala Municipality of Dar Es Salaam Region, Tanznia. East Afr J Public Health.

[20] Bertuzzo E, Mari L, Righetto L, Gatto M, Casagrandi R, Blokesch M (2011). Prediction of the spatial evolution and effects of control measures for the unfolding Haiti cholera outbreak. Geophys Res Lett.

[21] Ryan ET (2011). The cholera pandemic, still with us after half a century: time to rethink. PLoS Negl Trop Dis.

[22] Siddique AK, Ahmed S, Iqbal A, Sobhan A, Poddar G, Azim T (2011). Epidemiology of rotavirus and cholera in children aged less than five years in rural Bangladesh. J Health Popul Nutr.

[23] Advances in Thermal and Non-Thermal Food Preservation. Editor(s): Gaurav Tewari, Vijay K. Juneja. doi: 10.1002/9780470277898.

[24] Borroto RJ, Martinez-Piedra R (2000). Geographical patterns of cholera in Mexico, 1991–1996. Int J Epidemiol.

[25] Malavade S, Narvaez A, Mitra A, Ochoa T, Naik E, Sharma M (2011). Cholera in Ecuador: current relevance of past lessons learnt. J Glob Infect Dis.

[26] Swerdlow DL, Mintz ED, Rodriguez M, Tejada E, Ocampo C, Espejo L (1992). Waterborne transmission of epidemic cholera in Trujillo, Peru: lessons for a continent at risk. Lancet.

[27] Sasaki S, Suzuki H, Igarashi K, Tambatamba B, Mulenga P (2008). Spatial analysis of risk factor of cholera outbreak for 2003–2004 in a Peri-urban area of Lusaka, Zambia. Am J Trop Med Hyg.

[28] Alexander R, Benita A, Lucas K, Freda M, Berry R, Enoch P (2012). Cholera risk factors, Papua New Guinea. BMC Infec Dis.

[29] Ali M, Emch M, Donnay JP, Yunus M, Sack RB (2002). Identifying environmental risk factors for endemic cholera: a raster GIS approach. Health Place.

[30] Colombara DV, Cowgill KD, Faruque ASG (2013). Risk factors for severe cholera among children under five in rural and Urban Bangladesh, 2000–2008: a hospital-based surveillance study. PLoS One.

[31] Dunkle SE, Mba-Jonas A, Loharikar A, Fouché B, Peck M, Ayers T (2011). Epidemic cholera in a crowded urban environment, Port-au-Prince, Haiti. Emerg Infect Dis.

